# Supplementary material for: Probability-Density-Based Deep Learning Paradigm for the Fuzzy Design of Functional Metastructures
Source: Research (Wash D C). 2020 Sep 22;2020:8757403. doi: 10.34133/2020/8757403 (PMC7528036; doi:10.34133/2020/8757403)
Supplement: Supplementary Materials — Section S1: statistical theorem of probability-density-based networks. Section S2: technical details of the PDN architecture. Section S3: principal component analysis. Section S4: acoustic experiments on transmission measurement. Fig. S1: neural architecture of PDN. Fig. S2: additional examples for PDN-based reverse designs. Fig. S3: robustness of PDN towards similar target functionalities. Fig. S4: PDN versus direction interpolation. Fig. S5: acoustic functionalities of interpolated structures. Fig. S6: visualization of the principal component analysis. Fig. S7: acoustic standing wave tube. [file 8757403.f1.zip › Research_Supplementary information_final_1.docx]

**Supplementary Information**

**Probability-density-based deep learning for the fuzzy design of functional meta-structures**

Ying-Tao Luo1,a), Peng-Qi Li1,a), Dong-Ting Li2,a), Yu-Gui Peng1,3, Zhi-Guo Geng1, Shu-Huan Xie2, Yong Li2*, Andrea Alu3*, Jie Zhu4,5*, Xue-Feng Zhu1*

1 School of Physics and Innovative Institute, Huazhong University of Science and Technology, Wuhan, 430074, P. R. China.

2 Institute of Acoustics, Tongji University, Shanghai 200092, P. R. China.

3 Photonics Initiative, Advanced Science Research Center, City University of New York, 85 St. Nicholas Terrace, New York, NY 10031, USA

4 Department of Mechanical Engineering, Hong Kong Polytechnic University, Hong Kong SAR, P.R. China.

5 Hong Kong Polytechnic University Shenzhen Research Institute, Shenzhen 518057, P.R. China.

a)Y.-T. L., P.-Q. L. and D.-T. L. are equally contributed.

Requests for materials should be addressed to Y. L. (yongli@tongji.edu.cn), A. A. (aalu@gc.cuny.edu), J. Z. (jie.zhu@polyu.edu.hk), X.-F. Z. ([xfzhu@hust.edu.cn](mailto:xfzhu@hust.edu.cn)).

**Contents**

**Section S1. Statistical theorem of probability-density-based networks.**

**Section S2. Technical details of the PDN architecture.**

**Section S3. Principal component analysis.**

**Section S4. Acoustic experiments on transmission measurement**

**Section S1. Statistical theorem of probability-density-based networks**

In this section, we provide the theoretical background of the proposed probability-density-based network (PDN). As described by Eqs. (7)-(8) in the main text, the model’s optimization objective is described by the principle of maximum likelihood estimation (MLE). From the theoretical derivation of MLE, we can unequivocally demonstrate that PDN has a feasible optimization goal that makes up for the flaws in original artificial neural network in dealing with multivalued problems. Here we consider the dataset independently collected by numerical simulations from the joint distribution , where we denote

(S1)

where *X* denotes input features, *Y* denotes target labels, and *n* denotes the quantity of data samples. In terms of expression in the probabilistic graphical model, conditional probability and joint probability are equal to each other, since the marginal probability is always unitary. Thus, we have

(S2)

This joint distribution , or the conditional distribution , is the probability that we seek out to approximate, which represents the intrinsic physical relation between acoustic transmission and meta-structures. Although is unknown, the collected samples (*X*, *Y*) are independent and identically distributed, which thus defines the estimated distribution as an approximation. Our deep learning model with the parameter *θ* trained by the loss function of input variables *x* and label variables *y* is expected to approximate .

To design a feasible optimization goal, the Kullback-Leibler divergence (KLD), also known as the relative entropy, is introduced to measure the difference between and . Because PDN needs to approximate the estimated data distribution, naturally one possible optimization goal is to minimize the KLD, which is

(S3)From the derivation of minimizing KLD, we attain the same optimization goal that appeared in Eq. (8) of the main text. From the view of maximum likelihood, we can also derive the equation. In statistical inference, the likelihood function measures the fitness of a statistical model to data samples (*X*, *Y*) of the model parameters *θ*. The estimation function is used to choose the value of *θ* that maximizes the probability of observing (*X*, *Y*), which is expressed as follows

(S4)

Hereby, by maximizing the likelihood function, we obtain

(S5)

As the derivation of formula shows, the minimization of KLD is equivalent to the maximization of the likelihood function. Because logarithms are strictly increasing functions and the product of a large number of small probabilities may cause underflow, the likelihood maximization is replaced by the equivalent log-likelihood maximization in our case. Therefore, the optimization goal for *θ* is finally written as

(S6)

The maximum likelihood principle is universal to all statistical models, including neural networks. It should be noted that this statistical mechanism is feasible regardless of the specific type of data relation that the model is dealing with. The problem encountered in meta-structure inverse designs is the one-to-many feature-label relation. The reason why the original artificial neural network with mean square error as the regression loss fails to fit, is that one of its assumptions is invalidate for multivalued problems. The artificial neural network uses mean square error as the optimization goal

(S7)

The above equation with the estimated *y* as the output of our deep neural model can also be interpreted by the maximum likelihood principle. Assuming that the target label obeys the Gaussian distribution , we set the optimization goal to maximize the probabilistic density, which is equivalent to maximizing the sum of log-likelihood

(S8)

This least square estimation could only be feasible when the target label obeys a single normal distribution. However, due to the one-to-many relation in meta-structure inverse design, the target label cannot be regarded as a unimodal distribution but rather a multimodal distribution. Therefore, a neural network following the mean square error is trained to output the cumulative average of multiple labels. It is impossible to make to obey independent Gaussian distributions simultaneously and infer all the labels from the cumulative average, on condition that the estimation of target label is deterministic. Assuming , we would simply get

(S9)

How to design a multimodal distribution that is trainable? Firstly, it occurs to us that in quantum mechanics, a superposed state allows the likelihood of a particle in a given position or momentum to be determined. The superposition of multiple eigenstates is expressed by

(S10)

in which denotes a possible eigenstate of a quantum system.

In quantum mechanics, the dimension of is the probability magnitude. The probability of measuring a particle at position *x* is the modular square

(S11)

where is the interference term that represents quantum coherence. If we consider the von Neumann measurement scheme, the quantum states in an open quantum system evolve together with the environmental states, so that interference terms can be considered as trivial in a classical probability mixture scheme. Therefore, we have

(S12)

Coming back to the multivalued inverse design problem, we are interested in finding an analog of that could give all the plausible solutions. If we construct a system to do the reverse design in a similar way, *i.e.*, to use a probability mixture to describe the design output, we will solve the problem that is intractable for the mean square error of maximum likelihood principle. Recalling the intriguing concept of quantum collapse, the eventual state is not settled until the system interacts with the external world, or an “observation” happens. In PDN, the probabilistic sampling at rear ends mimics the “observation”, which makes the probability mixture collapse to a specific output of meta-structure. That is to say, if we make the “observation” by sampling, the scheme will return to the mean square error estimation, where each obeys a single Gaussian distribution . By separating modeling and “observation”, we show that PDN is actually equivalent to several ANNs with each ANN fitting a single label.

**Section S2. Technical details of the PDN architecture**

The PDN model is proposed on the level of statistics and optimization with a wide generality for its specific architectural design. For example, for tasks involving image or signal processing, a convolutional architecture is suitable; for language processing, a sequential recurrent architecture can also be efficient. As the meta-structure inverse design is hard to categorize, we use the fully connected layers that are featured with no inductive bias. One of the advantages of PDN is that, unlike deep generative models, it is directly designed to model a multivalued function and has a strong representation learning capability with stable optimization in inverse design. The neural architecture of PDN used for meta-structure inverse design is shown in Fig. S1.


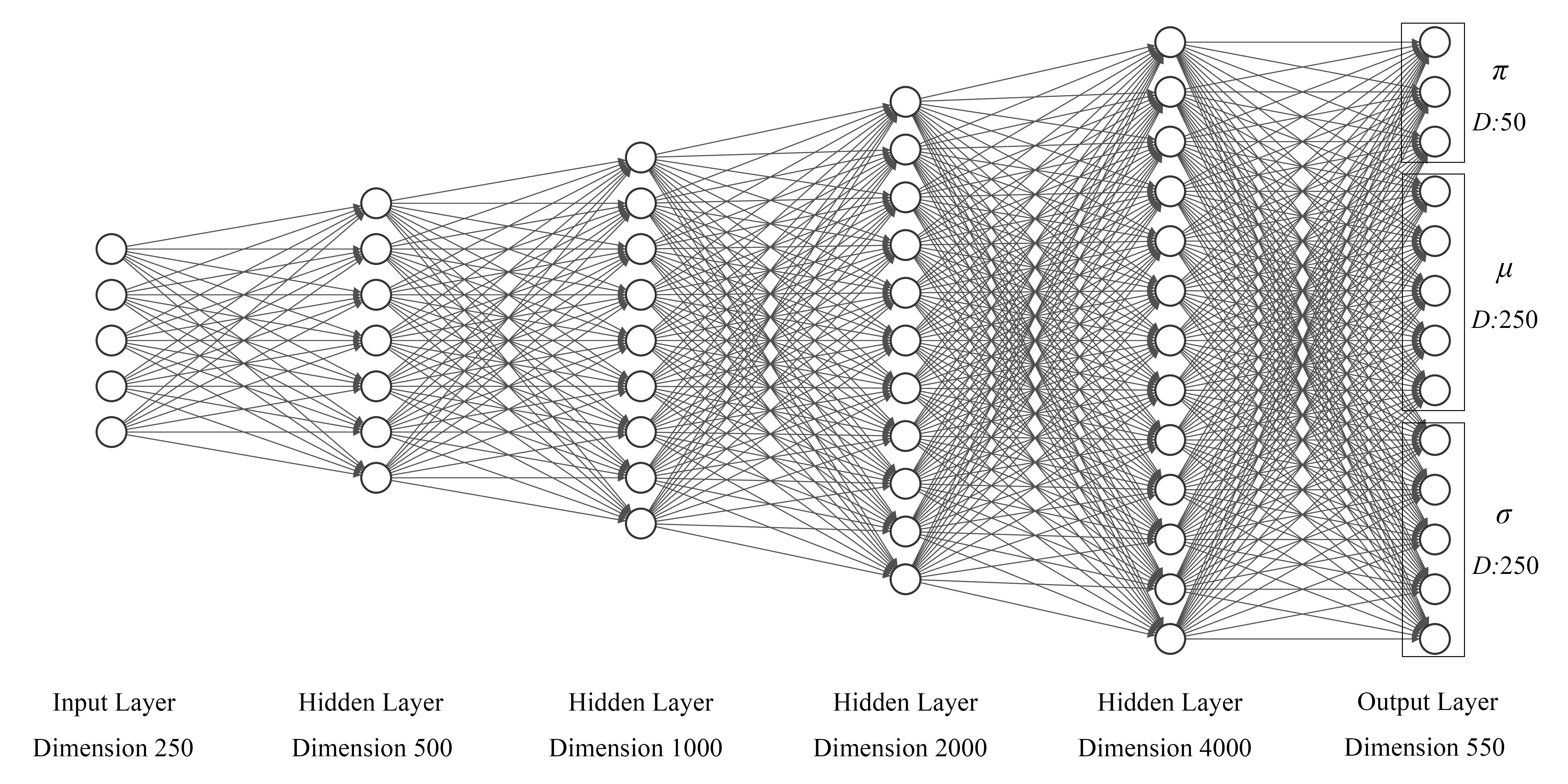


**Fig. S1.** **Neural architecture of PDN.** The neural architecture of PDN extracts high-dimensional features layer by layer by increasing the number of neurons. In the output layer, neurons are divided into three parts, corresponding to mixing coefficient, mean and deviation, respectively. Because the number of distributions is 50 and the output structural size is 5, the number of neurons for mean and deviation are calculated as 50×5=250. The output layer then constructs the probability mixture.

Although the theorem has been discussed in Section S1 and the main formula are given in the main text, there still remain some technical details that could affect the training and efficiency of the PDN model. The first problem is that it is impossible to have a priori knowledge on how many plausible meta-structures each transmission spectrum corresponds to for a multivalued inverse design problem. Although this does not affect the accuracy of prediction, the value of as a hyper-parameter is not able to be set appropriately without a priori assumption upon the number of superposed distributions. In practice, through previous investigation of the data, we could estimate for the 1-to-*m* relation and set the valueof manually, hopefully bigger than to ensure accuracy. Note that it is acceptable even if , because the scale factor can automatically mute the effects of unimportant redundant eigenstate by lowering its value. A bigger actually does not increase the computational complexity in a linear scale, as the number of parameters is mostly determined by the neurons of many previous layers. For example, in our case, the parameters of previous layers are nearly as big as 10 million, as calculated by multiplying the number of neurons of each layer and its successive layer, whereas the parameters of the last layer are at most 2 million. Moreover, as the inverse design is concerning multiple solutions to the desired functionalities, we could also determine from the numbers of the local maxima how many solutions we can find, and how likely it will deliver the desired functionalities.

To fulfill the optimization goals described in Eqs. (7)-(8) of the main text, PDN needs a learning criterion, *i.e.*, loss function, to measure the model’s convergence and provide effective gradients to update model weights *θ*. The loss function is provided by Eqs. (7)-(8) of the main text and expressed as follows

(S13)

The deep learning model is trained and evaluated by minimizing the loss function, which is a function of input *x*, target label *y* and weight parameters *θ*. This loss function, however, in possible schemes, may cause the training process to be blocked. Consider one of the schemes that as the training progresses, *σ* will gradually converge to 0. If the value of *σ* is not limited, due to the numerical precision in the computer, the *σ* will just be recorded as “0” that

(S14)

As a result, the loss function will change accordingly to

(S15)

The infinite loss can no longer provide effective gradients for model’s optimization and will shut down the ongoing training process utterly. Moreover, we need to consider the scheme when

(S16)

This turns the probabilistic density into infinity even if *σ* does not approach 0. To tackle the problem, we improve by adding a threshold to the loss as

(S17)


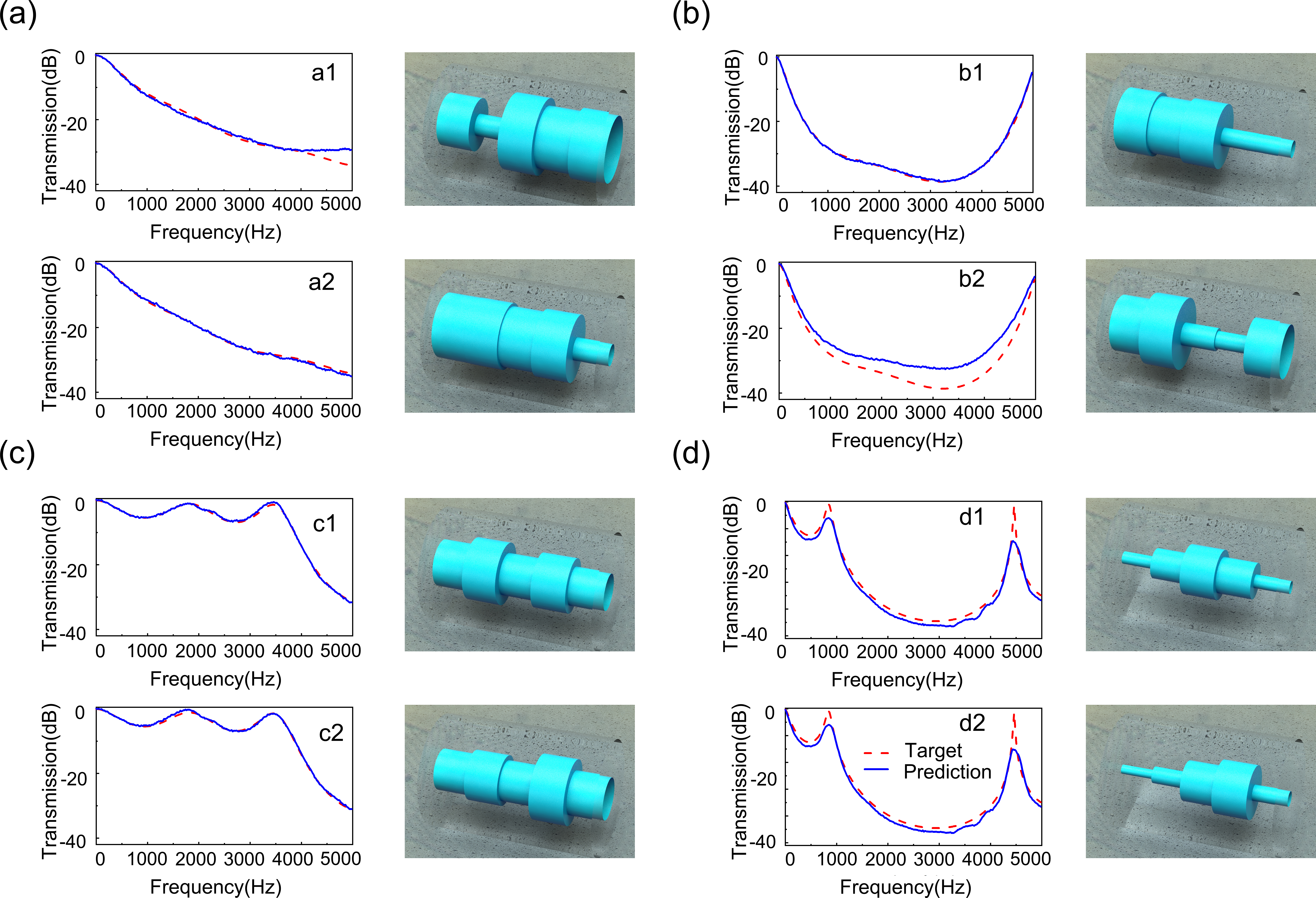


**Fig. S2.** **Additional examples for PDN-based reverse designs.** Targeting on different filtering functionalities, we use PDN to reversely design all best candidates. (a) Reverse design of anechoic meta-structures with a specific characteristic curve. (b) Reverse design of high-pass meta-structures with a specific characteristic curve. (c) Reverse design of low-pass meta-structures with a specific characteristic curve. (d) Reverse design of selective-band-pass meta-structures with a specific characteristic curve.

Besides the neural architecture and loss function, we also show the stability and effectiveness of PDN with more inverse design examples in Fig. S2, where the PDN presents strong generalizability and robustness. In Figs. S2(a)-(d), four different cases are taken into consideration, *i.e.*, reversely designing anechoic, high-pass, low-pass and selective-band-pass meta-structures for four different target characteristic curves. The results show a good agreement between the predicted curves of meta-structures and the target characteristic curves. In Fig. S2(d), we find that the predicted transmission in the pass bands is lower than the target one, yet the position of pass bands perfectly matches with the target. This can be explained by that in the optimization of PDN, the hyper-parameters are chosen for the best fitting of overall transmission. To tackle this problem, we can alter the original loss and increase the weight of peak points at the expense of global fitness.





**Fig. S3.** **Robustness of PDN towards similar target functionalities.** (a), (b) and (c) Three target transmission spectra with similarities, and all of the reversely designed meta-structures. Despite similarity exists in the three target transmission spectra, PDN can predict the meta-structures with very different geometries, which shows that PDN can capture even slight differences of the desired functionalities and can distinguish them in the reverse design.

In Fig. S3, we further show that PDN can capture the slight differences of desired functionalities in the fuzzy design of meta-structures. Here, the PDN solves a dilemma: even humans may have a hard time on defining two similar functionalities as trivial errors or completely different ones related to very unlike meta-structures. Previous deep learning models, such as ANN, simply regard this as errors. PDN can’t get fooled by similar desired functionalities with the output always the same ones. For example, in Figs. S3(a)-(c), despite the target transmission spectra look very similar in shapes with only slight differences, PDN outputs very different meta-structures nonetheless. It is a strong evidence that PDN does not confuse target transmission spectra just due to their similarity. This robustness is vital especially when defending adversarial samples. For example, the predicted structures in Fig. S3(a) does not resemble any of the predicted structures in Figs. S3(b)-(c). It shows that PDN considers uncertainty in data modeling and reports all the plausible structures without missing. Finally yet importantly, it should be noted that the transmission spectra we are exploring in the on-demand design have the same level of local feature complexity with the labeled samples in datasets. More complicated transmission curves can be implemented via meta-structures with more layers inside, where PDN is still workable.

Although we show that PDN can generalize and is not a simple look-up search in the training dataset, one might argue that maybe interpolations in the training data can get us competitive results. After all, it is in line with the smoothness hypothesis that perhaps a desired transmission lying in between two close transmissions in the training dataset can be attained by directly interpolating in corresponding structures. However, by experiments, we show that interpolation cannot get competitive results. In Fig.S4, we present the transmissions of two close structures, namely Structure **A** and Structure **B**, and the transmissions of PDN interpolation and direct average interpolation. As can be seen, in Figs. S4(a)-(b), PDN interpolations are unlike direction interpolations; only in one case of Fig. S4(c), PDN interpolation is similar to the direction interpolation. This shows us that the generalization of PDN to the interpolated areas is not a simple average calculation, but a comprehensive modeling of the inverse physical relation.


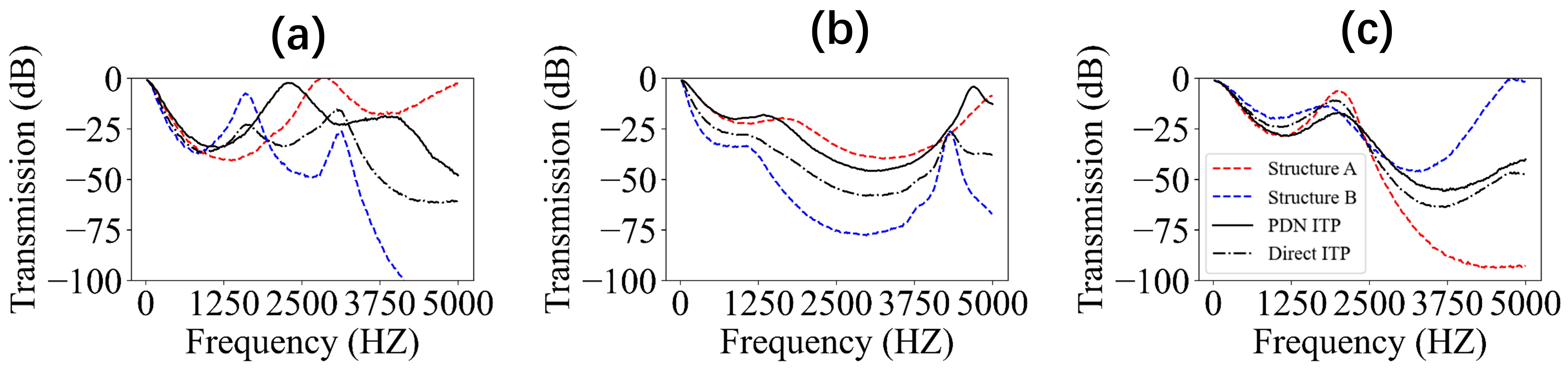


**Fig. S4. PDN versus direction interpolation.** Here three examples are shown in each subfigure. PDN ITP denotes the transmission of PDN predicted structure, while direct ITP denotes the average transmission of Structures **A** and **B**, *i.e.*, their interpolation. (a) Structure **A** (10.875 mm, 5.438 mm, 5.438 mm, 5.438 mm, 7.25 mm), **B** (9.969 mm, 6.344 mm, 5.438 mm, 6.344 mm, 6.344 mm); (b) Structure **A** (1.813 mm, 5.438 mm, 7.25 mm, 9.969 mm, 3.625 mm), **B** (2.719 mm, 6.344 mm, 6.344 mm, 10.875 mm, 4.531 mm); (c) Structure **A** (14.5 mm, 7.25 mm, 12.688 mm, 5.438 mm, 10.875 mm), **B** (13.594 mm, 8.156 mm, 12.688 mm, 6.344 mm, 9.969 mm)

In Fig. S5, the three examples exhibit acoustic functionalities of different structures where the interpolations of training data points on PDN reflect the change from one to another. This change is unpredictable, as shown in Fig. S5(a1) that the interpolations of the first layer, the third layer and the fifth layer present very different transmissions, and it is even harder to tell which layer contributes most to the change when all layers are interpolated. For example, in Fig. S5(a1), negative interpolation at the first layer of radius 2.984 mm, which is 2.984 – 0.90625 = 2.07775 mm, shows a movement down below the ground truth transmission, while the positive interpolation of 2.984 + 0.90625 = 3.89025 mm moves upwards of the transmission curve. The changes caused by the interpolation do not share a common direction. For example, in Figs. S5(b1) and (c1), the negative interpolations do not present changes down below the ground truths like the case in Fig. S5(a1), but move upwards. Simple interpolations can hardly help in searching for the target structures that have the desired acoustic functionalities. These prove that PDN is essential and powerful in inverse design.





**Fig. S5. Acoustic functionalities of interpolated structures.** Here three ground truth structures from the training dataset are set: (a) S1 [2.984 mm, 4.176 mm, 6.197 mm, 6.846 mm, 8.649 mm], (b) S2 [10.875 mm, 9.062 mm, 5.437 mm, 7.250 mm, 12.687 mm] and (c) S3 [12.6875 mm, 3.625 mm, 5.4375 mm, 3.625 mm, 9.0625 mm]. Transmission spectra of the interpolated structures with ±0.90625 mm deviation are obtained: (a1)-(a4) S1 with the deviation at the first layer, the third layer, the fifth layer and all five layers, respectively; (b1)-(b4) S2 likewise; (c1)-(c4) S3 likewise.

**Section S3. Principal component analysis**

Using the technique of principal component analysis (PCA), we could reduce the 5D mixed probability distribution down to a 2D one for visualization. The idea of PCA is to extract important information from original data and present it by fewer orthogonal variables. The choice of new orthogonal coordinate axes reveals the pattern of similarity of the original variables, which follows the principle that each selected axis maximizes the variance in the plane orthogonal to other axes. There are basically two ways to get the principal component axes containing maximum difference, which are the eigen-decomposition and the singular value decomposition (SVD) of covariance data matrix, respectively.

Here we consider dataset *X* with *n* samples, and each sample has *h* features

(S18)

where *X* can be represented by column feature vector or row data vector . The goal of PCA is to maintain the principal information and minimize the error that is introduced by the dimensional reduction, for example, reducing the dimensions from *h* to *k*. We can use a set of standard unit orthogonal basis, a column vector, to define the new axes that PCA selects

(S19)

With the projection direction, the projection of a data point would be . The error of cutting this dimension for dataset *X* is

(S20)

To realize the *h*-to-*k* dimension reduction, we ought to minimize *J*

(S21)

Using Lagrange multiplier, we could instead minimize

(S22)

Then, we take the derivative of Eq. (S22) to find where the extreme value locates

(S23)

Note that this equation follows the exact form of eigen-decomposition. If we return this term to the error function, the minimized error would be the eigenvalue

(S24)

Naturally, the minimization of error is determined by the eigenvalue of each feature dimension. We could use the eigen-decomposition method to get eigenvalues, and discard the eigenvectors that correspond to the eigenvalue minimums.

After normalization, the mean of each feature column is equal to zero

(S25)

We can then calculate the covariance matrix, a square matrix of order *h*, as follows

(S26)

If all eigenvectors are linearly independent, can be diagonalized as

(S27)

where *Q* is the eigenvector and denote the diagonalized eigenvalues. To get each eigenvector *y* and eigenvalue *λ* of , we can solve the equation

(S28)

After finding all the solvers, we concatenate all the eigenvalues and eigenvectors into matrices, respectively. By concatenating the eigenvectors corresponding to the largest eigenvalues, we can form the transformation matrix *P* with the shape (*k*, *h*). Finally, we can calculate the desired matrix that reduces the original feature dimensions from *h* to *k* and maintains the principal information via

(S29)

Alternatively, using SVD to replace eigen-decomposition, we can also calculate valid eigenvectors and eigenvalues to reduce the dimensions. SVD is a generalization of spectral analysis theory on arbitrary matrices, which has a wider applicability. The basic formula of SVD is described as

(S30)

where *U* is the left singular vector, *i.e.*, a unitary square matrix of order *n*. is the right singular vector, *i.e.*, the conjugate transpose matrix of order *h*. *S* is a diagonal matrix of nonnegative real numbers. Therefore, . The values of left and right singular vector are determined by eigen-decomposition

(S31)

where the columns of *V* are the compressed orthogonal basis, *i.e.*, the eigenvector of . As we recall from Eq. (S27), the eigen-decomposition is . On the other hand, from the perspective of SVD, . It can be seen that the eigen-decomposition form and the SVD form are consistent with each other in the forms of and . The minimization of error is determined by the eigenvalue of each feature dimension. We could use the eigen-decomposition method to get eigenvalues, and discard the eigenvectors that correspond to the eigenvalue minima.

To help readers understand how PCA works, we visualize the case of reducing the data dimensions from 2 to 1 in Fig. S6. Here, features *x* and *y* are the only two variables, while the principal component is the compressed new axis that is chosen by PCA algorithm. The data points used in this case are normally distributed along *y=x*. As shown in Fig. S6, the data samples represented by the black hollow circles are projected onto the red principal component axis by PCA algorithm. Along the blue compressed axis, the overall projection error is minimal, indicating that the reduced dimension is rather trivial compared to the principal component.


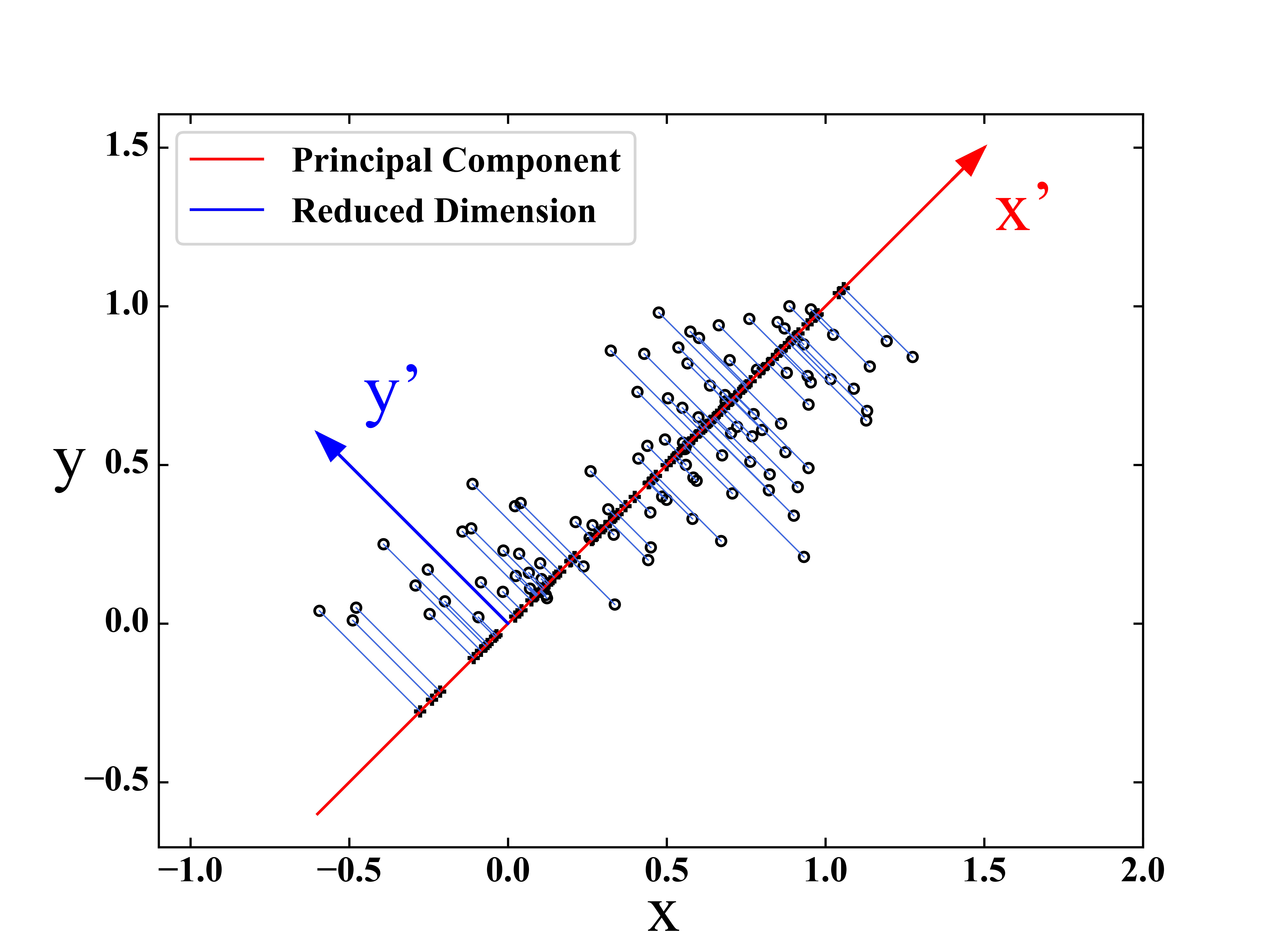


**Fig. S6. Visualization of principal component analysis.** According to the distribution of data samples represented by black hollow circles, the PCA algorithm chooses the principal component axis  by SVD or eigen-decomposition that minimizes the error of dimension reduction along the reduced orthogonal axis.

**Section S4. Acoustic experiments on transmission measurement**

In this section, we show the photograph of a commercial acoustic standing wave tube that we used in the transmission spectrum measurement, as shown in Fig. S7.


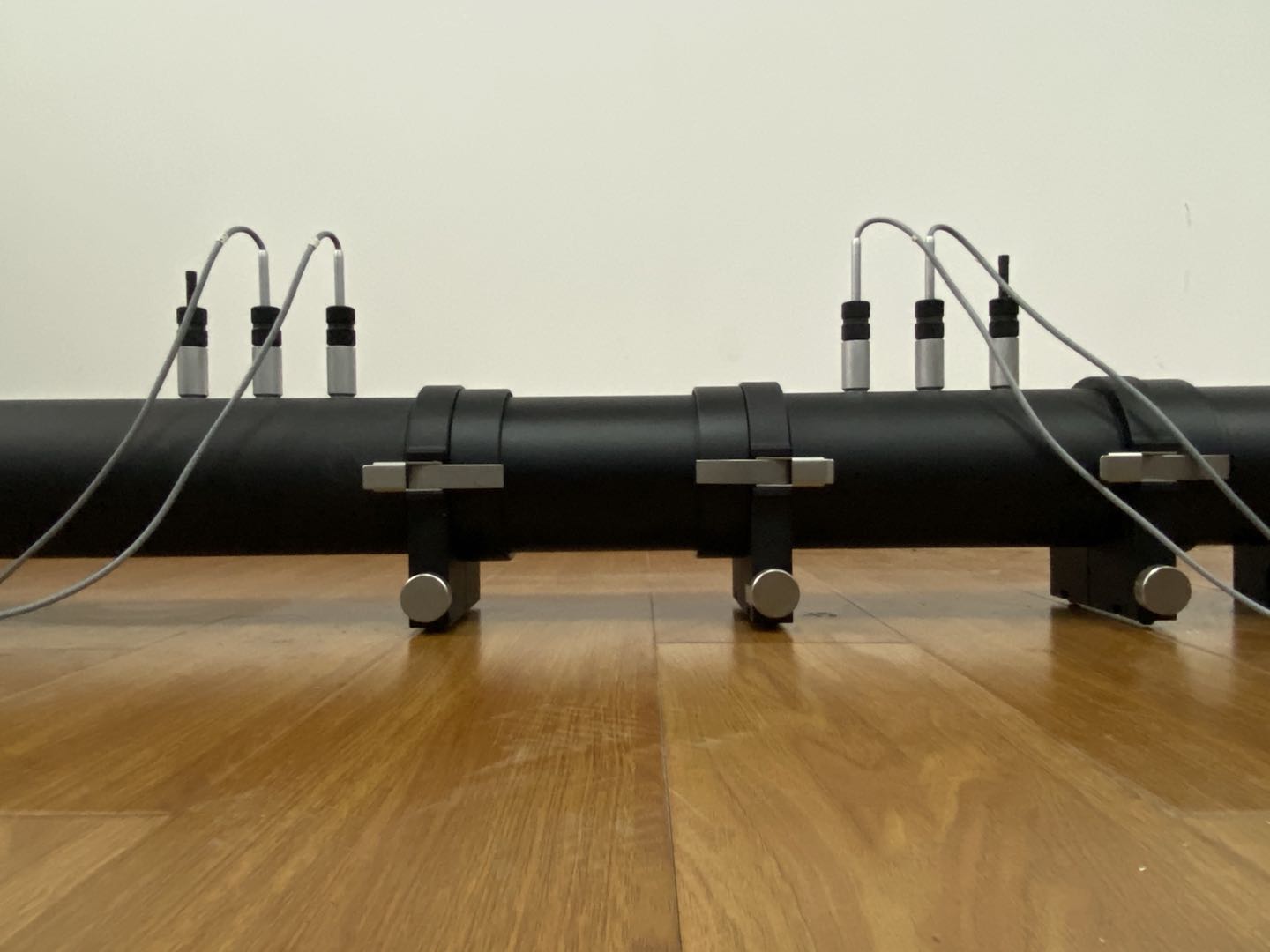


**Fig. S7. Acoustic standing wave tube.** The device type is Brüel & Kjær type-4206T.
